# Characterization of Polyhydroxyalkanoates Produced at Pilot Scale From Different Organic Wastes

**DOI:** 10.3389/fbioe.2021.628719

**Published:** 2021-02-18

**Authors:** Laura Lorini, Andrea Martinelli, Giorgio Capuani, Nicola Frison, Maria Reis, Bruno Sommer Ferreira, Marianna Villano, Mauro Majone, Francesco Valentino

**Affiliations:** ^1^Department of Chemistry, University of Rome La Sapienza, Rome, Italy; ^2^Department of Biotechnology, University of Verona, Verona, Italy; ^3^Department of Chemistry, Nova University of Lisbon, Lisbon, Portugal; ^4^Biotrend SA, Biocant Park, Cantanhede, Portugal; ^5^Department of Environmental Science, Informatics and Statistics, “Ca Foscari” University of Venice, Venice, Italy

**Keywords:** polyhydroxyalakanoates, mixed microbial culture (MMC), thermal properties, molecular weight, urban waste

## Abstract

Polyhydroxyalkanoates (PHAs) production at pilot scale has been recently investigated and carried out exploiting different process configurations and organic wastes. More in detail, three pilot platforms, in Treviso (North-East of Italy), Carbonera (North-East of Italy) and Lisbon, produced PHAs by open mixed microbial cultures (MMCs) and different organic waste streams: organic fraction of municipal solid waste and sewage sludge (OFMSW-WAS), cellulosic primary sludge (CPS), and fruit waste (FW), respectively. In this context, two stabilization methods have been applied, and compared, for preserving the amount of PHA inside the cells: thermal drying and wet acidification of the biomass at the end of PHA accumulation process. Afterward, polymer has been extracted following an optimized method based on aqueous-phase inorganic reagents. Several PHA samples were then characterized to determine PHA purity, chemical composition, molecular weight, and thermal properties. The polymer contained two types of monomers, namely 3-hydroxybutyrate (3HB) and 3-hydroxyvalerate (3HV) at a relative percentage of 92.6–79.8 and 7.4–20.2 w/w, respectively, for Treviso and Lisbon plants. On the other hand, an opposite range was found for 3HB and 3HV monomers of PHA from Carbonera, which is 44.0–13.0 and 56.0–87.0 w/w, respectively. PHA extracted from wet-acidified biomass had generally higher viscosity average molecular weights (*M*_*v*_) (on average 424.8 ± 20.6 and 224.9 ± 21.9 KDa, respectively, for Treviso and Lisbon) while PHA recovered from thermally stabilized dried biomass had a three-fold lower *M*_*v*_.

## Introduction

Among different kinds of biopolymers, polyhydroxyalkanoates (PHAs) are one of the most interesting and promising thanks to their mechanical properties and biodegradability. As an alternative to industrial scale production based on pure culture (Gholami et al., 2016), the application of low-cost substrates based on organic wastes and by-products is of an extreme interest, specially coupled with the use of mixed microbial cultures (MMCs) that do not require sterile conditions or excessive operation control (Serafim et al., 2008a; Nikodinovic-Runic et al., 2013; Echegoyen et al., 2017). In fact, it is possible to combine the use of both MMCs (Beccari et al., 1998; Dionisi et al., 2004) and economic feedstocks (Dionisi et al., 2005; Bengtsson et al., 2008; Albuquerque et al., 2010; Bengtsson et al., 2010b; Campanari et al., 2014). Recently, the conversion of both wastewaters and volatile fatty acids (VFAs) mixtures obtained from the fermentation of different organic feedstocks, by MMC, have been investigated for the implementation of such a three-step process at pilot scale, by the integration of MMC-PHA production to wastewater treatment (Valentino et al., 2019; Conca et al., 2020; Moretto et al., 2020). However, downstream processing, including PHA-rich biomass stabilization and polymer extraction, are among the most important factors affecting the overall PHA production cost and need to be optimized for pilot scale. The most studied methods for recovering PHAs have been summarized in recent reviews (Kosseva and Rusbandi, 2018; Pérez-Rivero et al., 2019) and can be grouped into two main categories: solvent extraction and digestion of the NPCM (non-PHA cellular material). Up to now, regarding the MMC PHA-rich biomass, the use of several non-chlorinated solvents (Werker et al., 2014; Samorì et al., 2015) and, more recently, the disruption of NPCM through chemical agents (Burniol-Figols et al., 2020) and surfactants (Colombo et al., 2020) have been investigated. In the view of MMC-PHA production at industrial scale and a subsequent market scenario, specific chemo-mechanical and thermal properties of the polymer need to be controlled. In a recent study, two types of stabilization method have been applied at pilot scale on PHA obtained from organic wastes, in order to preserve the amount of PHA produced after the accumulation step and its properties (Lorini et al., 2020). The recovered polymer was extensively characterized and resulted in good thermal and chemical properties, while it showed variable molecular weight (between 100 and 450 kDa) dependent on the applied stabilization method. Indeed, molecular weight should be higher than 200 kDa and it is influenced by the PHA production process and downstream, including the extraction step (Laycock et al., 2014b). The availability in literature regarding the chemo-mechanical and thermal characterization of MMC-PHAs is very limited, however, the published studies highlighted an increase in melting temperature (*T*_*m*_) for MMC-PHA respect to pure culture derived PHA (Bengtsson et al., 2010b) and a similar decomposition temperatures (270°C) to the commercial poly-3-hydroxybutyrate-co-3-hydroxyvalerate [P(3HB-co-3HV)] (Carrasco et al., 2006). Furthermore, monomeric composition, i.e., 3HV content, showed to have effects on different polymer properties (Arcos-Hernández et al., 2013; Lorini et al., 2020), such as crystallinity.

Thus, the evaluation of the quality of waste-derived PHA and the comparison with the properties of traditional plastics are necessary for commercial purposes and they must meet the standards required for various applications. Furthermore, for the scaling up of MMC-PHA production it is important to optimize an extractive protocol in order to minimize the overall production costs. On the other hand, the concept of novel biorefineries for urban waste valorization allows to integrate MMC–PHA production with wastewater treatment and other biowaste collection, reducing the production costs and the environmental impact. In this regard, the present study shows the results of a wide characterization, including thermal and chemical properties, of several samples of three grades of PHAs, produced by three pilot plants for PHA production from biowaste and recovered with the same extractive method (based on the use of aqueous-phase inorganic reagents). Specifically, the three pilot units produced PHA from different organic feedstocks: the organic fraction of municipal solid waste (OFMSW) and waste activated sludge (WAS) in Treviso plant; fruit waste in Lisbon; cellulosic primary sludge (CPS) in Carbonera.

## Materials and Methods

The effects of an extraction method based on aqueous-phase inorganic reagents were evaluated on PHAs recovered from different biomasses, coming from three pilot plants using three types of feedstock, named TV-samples (from Treviso, OFMSW-WAS), L-samples (from Lisbon, FW), and C-samples (from Carbonera, CPS).

### PHA From Treviso Pilot Plant

Within the pilot platform of Treviso (northeast Italy), the PHA was produced from a feedstock composed by a mixture of the liquid slurry coming from squeezed OFMSW and WAS from the treatment of municipal wastewater. For the sake of simplicity, this feedstock mixture is indicated as OFMSW-WAS. The latter was characterized by a volatile/total solids ratio of 80 ± 1% (VS/TS); a soluble chemical oxygen demand (COD_*SOL*_) of 22 ± 1 (gCOD L^–1^); total Kjeldahl nitrogen (TKN) equal to 29 ± 3 (g N kg^–1^ TS) and total phosphorus (P) equal to 2.3 ± 0.1 (g P kg^–1^ TS) (Moretto et al., 2020). The main process setup and operative conditions are extensively described in Valentino et al. (2019) and Moretto et al. (2020). The pilot plant consisted in a first anaerobic fermentation reactor (380 L) for PHA-precursors production (VFA), a second aerobic reactor (sequencing batch reactor, SBR; 100 L) for biomass cultivation, and a third fed-batch aerobic reactor (70–90 L) for PHA accumulation inside the cells (40–50% wt). This PHA-rich biomass (raw biomass) was collected following two different protocols of biomass stabilization, both addressed to the long-term PHA conservation inside the cells before the extraction/purification steps.

Following the first protocol, at the end of each accumulation, the PHA-rich biomass was left to settle under gravity and then the thickened slurry was centrifuged in a Heraeus Megafuge 40 with a Swinging Bucket Rotor (maximum radius: 195 mm; minimum radius: 83 mm) from Thermo Fisher Scientific (Waltham, MA, United States) for 15 min at 4,500 rpm. The wet pellet was thermally pre-treated at 145°C for 15 min, and then dried overnight in the same oven at 60°C (sample TV20).

In the second protocol, at the end of each accumulation, the PHA-rich biomass was acidified with H_2_SO_4_ down to pH 2.0, and left to settle under gravity overnight. The thickened slurry was then centrifuged in a Heraeus Megafuge 40 with a Swinging Bucket Rotor (maximum radius: 195 mm; minimum radius: 83 mm) from Thermo Fisher Scientific (Waltham, MA, United States) for 15 min at 4,500 rpm. Finally, the wet pellet was stored in the fridge (4°C) (samples TV1-12).

### PHA From Lisbon Pilot Plant

The concept of PHA production process is similar to that previously described. The main difference is the kind of the feedstock, which in this case was organic waste coming from fruit processing. The FW was characterized by a high VS/TS ratio (98.0 ± 2.27%); a COD_*SOL*_ equal to 3.76 gCOD/L; very low ammonia (N-NH_4_^+^) and phosphate (P-PO_4_^3–^) concentration, 1.26 ± 0.07 mg N/L and 2.39 ± 0.67 mg P/L, respectively. The pilot units in Lisbon’s facility consists in three reactors: an upflow anaerobic sludge bioreactor (UASB) with a volume of 60 L, a sequential batch reactor (SBR) with a volume of 100 L and a stirring tank reactor operating in fed-batch mode with 60 L of volume. Fermented FW was the feedstock for both biomass cultivation and PHA accumulation steps. The selected biomass was able to accumulate the polymer up to 70% of its cell dry weight. At the end of accumulation operation, the second stabilization protocol (described in section “PHA From Treviso Pilot Plant”) was applied: the reactor slurry was settled to remove as much water as possible and the biomass was acidified down to pH of 2.0–2.5, in order to prevent polymer degradation. The PHA-rich biomass was then stored in a fridge (4°C) before extraction.

### PHA From Carbonera Pilot Plant

The pilot plant located in Carbonera wastewater treatment plant (WWTP) (Treviso, Italy) integrated the via-nitrite nitrogen removal with PHAs production from the sidestream of Carbonera WWTP (Treviso, Italy). A novel substrate derived from fermentation of CPS was used as carbon source to evaluate the impacts on PHAs production and it was characterized by a VS/TS ratio equal to 92%; a relatively high content in terms of COD (1.5 g O_2_/g TS) and a low content of TKN (16/22 mg N/g TS) and P (1.7/4.7 mg P/g TS) (Da Ros et al., 2020). Process setup and operative conditions are extensively described in Conca et al. (2020). The pilot plant comprised the following units: i) rotating belt dynamic filter (RBDF) for the recovery of CPS; ii) fermentation unit for the production of VFAs; iii) ultrafiltration unit (UF) for solid/liquid separation of the fermented sludge; iv) nitritation sequencing batch reactor (N-SBR) for the oxidation of ammonia to nitrite; v) selection SBR (S-SBR) where aerobic-feast and anoxic-famine conditions were established to select PHA-accumulating biomass; and vi) an accumulation SBR (A-SBR) were intracellular PHA content was maximized through the feed-on-demand strategy. The S-SBR consisted in a stainless-steel SBR with a total volume of 2.8 m^3^ and operated under (aerobic)-feast and (anoxic)-famine conditions. The accumulation reactor (A-SBR) consisted in a stainless-steel tank with a volume of 1 m^3^ which operated as a fed-batch reactor. At the end of accumulation operation, the second stabilization protocol (described in section “PHA From Treviso Pilot Plant”) was applied: the reactor slurry was settled to remove as much water as possible and the biomass was acidified down to pH of 2.0–2.5, in order to prevent polymer degradation. The PHA-rich biomass was then stored in a fridge (4°C) before extraction.

### PHA Extraction

#### Aqueous Phase Inorganic Reagents (Reserved Protocol)

An extraction method involving a mixture of aqueous phase inorganic reagents has been optimized by Biotrend S. A. for PHA recovery from the cellular material of enriched biomass. The optimized method was carried out on PHA-rich biomasses coming from the three previously described pilot plants, stabilized at the end of the accumulation step following the thermal (sample TV20) and the acidification protocol (samples TV 1-12; L-samples; C-samples). At the end of the extraction, the polymer was dried obtaining a white powder. The optimized extraction method is reserved by Biotrend S. A. and under patent application procedure.

### PHA Characterization

#### PHA Content and Composition Determination (GC-FID)

Approximately 3.5 mg of dried biomass or 5.0 mL of re-suspended samples (wet and dried thermally treated biomasses) were suspended in 2 mL of acidified methanol solution (at 3% v/v H_2_SO_4_) containing benzoic acid (at 0.005% w/v) as internal standard and 1 mL of chloroform in a screw-capped test tube. Then, an acid-catalyzed methanolysis of the PHA occurred and the released methyl esters were quantified by gas-chromatography (GC-FID Perkin Elmer 8410) according to the method described in Braunegg et al. (1978). The relative abundance of 3HB and 3HV monomers was determined using a commercial P(3HB-co-3HV) copolymer with a 3HV content of 5 wt. % (Sigma–Aldrich, Milan, Italy) as reference standard. The 3HV content in PHA was calculated as the ratio of 3HV and (3HB + 3HV) monomers (as wt. %).

#### Capillary Viscosimetry

A volume of 25 mL of PHA solution at the concentration of 0.5% w/v in chloroform were prepared for all the samples of extracted polymer. The equipment for the measure of the gravimetric flow time of each solution was composed by a SCHOTT AVS 350 viscosimeter with AVS/SHT sensor, a LAUDA CD15 thermostatic bath (working at 30°C) and a SCHOTT GERÄTE Ubbelohde capillary viscosimeter (ID = 0.46 mm). For each sample, 15 mL of solution 0.5% w/v were transferred in the Ubbelohde capillary viscosimeter and the flow time was measured through the optical sensor. Then, at least four dilutions were made directly in the viscosimeter by adding predetermined aliquots of solvent. From the flow time of each solution the intrinsic viscosity [η] of polymer samples was determined. The viscosity average molecular weight (*M*_*v*_) was calculated according to the Mark–Houwink equation:

(1) $$M_{v}\to \left[\eta \right]=K\cdot M_{v}^{\alpha }$$

with *K* = 7.7 × 10^–5^ e α = 0.82 (Marchessault et al., 1970)

#### Thermogravimetric Analysis (TGA)

The thermal stability of the samples was evaluated by TGA using a Mettler TG50 thermobalance. Approximately 5 mg of dried samples were weighted on the balance. The analysis was conducted in nitrogen flow (20 mL min^–1^) by heating the samples from 30 to 500°C at 10°C min^–1^.

#### Differential Scanning Calorimetry (DSC)

The thermal properties of the PHA samples were characterized by a differential scanning calorimeter Mettler Toledo DSC 822e. All the experiments were carried out under N_2_ flux (30 mL min^–1^) on about 3–6 mg of polymer, weighted in aluminum pans. TV-samples were analyzed applying a single heating scan at 10°C min^–1^ from room temperature to 190°C, in order to evaluate the real thermal behavior of the final product.

The sample crystallinity (*X*_*c*_) was evaluated from the equation:

(2) $$X_{c}=100\cdot \Delta \,H_{m}\,/\,\left(\Delta \,H_{m}^{0}\cdot \mathrm{purity}\right)$$

where Δ*H*_*m*_ is the melting enthalpy obtained from the single DSC heating scan and Δ*H*_*m*_^0^ = 146 J g^–1^ is the enthalpy of fusion of 100% crystalline PHB sample (Barham et al., 1984).

On the contrary, in order to evaluate the intrinsic thermal properties, the following temperature program was selected for the analysis of C-samples:

1. a heating scan at 10°C min^–1^ from RT to 190°C;
2. a rapid cooling at 30°C min^–1^ from 190 to −70°C;
3. a second heating up to 190°C at 10°C min^–1^.

The first heating is necessary to erase all the previous sample history.

#### PCA Analysis

Principal component analysis (PCA) was conducted using the software The Unscrambler ver. 10.5 (CAMO Software AS, Oslo, Norway).

## Results

Three grades PHA produced from three different organic wastes have been extracted and characterized. For the sake of a deeper discussion, the characteristics of fermented streams and the results in terms of overall PHA production yield of the three pilot plants are here reported. Within the pilot plant located in Treviso, the fermented mixture of OFMSW – WAS showed a predominance of acetic acid (24–34%) and propionic acid (10–20%), along with butyric acid (30–40%) and caproic acid (25–35%) fed to both selection and accumulation steps for PHA production. The overall process yield was calculated and resulted of 110 g PHA/kg VS (Moretto et al., 2020). On the other hand, pilot plant in Lisbon produced a fermented stream, starting from FW, characterized by a predominance of butyric and acetic acid, along with propionic acid, valeric acid, lactic acid and ethanol, with a resulting fermentative products concentration of 21.2 ± 1.6 gCOD L^–1^. As for Treviso process, the fermented stream fed both the selection and accumulation steps. It was estimated that 37.4 L of FW are necessary to produce 1.0 kg of PHA and then the resulted global yield of conversion was 26% on COD basis (*data under submission*). Finally, the fermentation of CPS in Carbonera pilot plant led to a fermented stream characterized by the presence of acetic, propionic, butyric and valeric acid and a quite high molar ratio between even and odd VFAs [0.49–0.52% mol/mol C_3_/(C_2_ + C_3_)]. The fermented stream CPS fed both the selection and the accumulation steps. As a result, the PHA production represented up to 17.5% of the COD conversion and a potential PHA production of 1.2 kg PHA/PE year was calculated (Conca et al., 2020).

### Purity and Monomeric Composition

Results obtained by GC-FID analysis conducted on extracted samples (Table 1) showed that the optimized extraction method allowed to reach very high purity, with an average value of 103.4 ± 2.1% w/w. The high PHA content in the recovered material was confirmed through TGA analysis. In fact the PHA content (% w/w), that indicates the purity of the sample, showed an average value of 98.9 ± 0.7% w/w. Sample TV20, extracted from a thermal stabilized biomass, had the lowest purity (85.5 ± 3.6% w/w). In Table 1, the 3HV content quantified from GC-FID analysis is also reported. It is possible to notice that samples from OFMSW-WAS and from FW are characterized by a 3HV content ranging between 16.3 and 19.1% w/w, with some exceptions. More in detail, samples TV20, L1, L2, and L3 (having 3HV content between 7.4 and 7.7% w/w) have been produced during the first operative year, while the remaining samples have been produced during the following operative years. In both cases, the two pilot platforms in Treviso and in Lisbon were operated in parallel, in order to obtain polymers with the same monomeric composition. Indeed, VFAs composition of the fermented mixtures, characterized by the predominance of even VFAs, led to a high 3HB percentage. On the other hand, a higher variability and an opposite trend were found for C-samples, in fact 3HV content is higher than 3HB, varying from 56 to 87% w/w, as a direct consequence of the high odd VFAs content in the feedstock (Albuquerque et al., 2011).

**TABLE 1:** Chemical and thermal properties of PHA samples produced in Treviso, Lisbon, and Carbonera pilot plants.

Sample	Feedstock	Pretreatment	Extraction	GC Purity (PHA % w/w)	TGA Purity (PHA % w/w)	$T_{\max}^{d}$ (°C)	$T_{10\%}^{d}$ (°C)	HV (% w/w)	References
*TV20				85.5 ± 3.6	86	307	289	7.67	
					
TV1				92.8 ± 1.9	100	274	249	18.9	
					
TV2				95.7 ± 0.6	100	299	274	18.3	
					
TV3				98.5 ± 3.8	100	295	271	18.6	
					
TV4				99.2 ± 2.4	100	284	260	18.4	
					
TV5	Treviso (OFMSW-WAS)			92.5 ± 1.3	98	278	253	18.1	
					
TV6				105.1 ± 1.2	100	276	251	18.9	
					
TV7				97.4 ± 1.6	100	293	267	18.2	
					
TV8				108.4 ± 1.3	100	290	264	19.1	
					
TV9				95.8 ± 3.9	100	289	270	16.3	
					
TV10				99.3 ± 0.1	100	285	264	16.3	
					
TV11				99.4 ± 1.1	100	285	263	18.8	
					
TV12		Wet acidification	Optimized aqueous-phase inorganic extraction method	99.7 ± 3.1	99	290	268	18.6	This study
					
L1				100.3 ± 3.1	94.6	270	247	7.7	
					
L2				100.9 ± 1.3	98.5	302	286	7.4	
					
L3				92.0 ± 0.3	93.7	272	254	7.4	
					
L4	Lisbon (FW)			116.1 ± 4.0	94.1	272	258	17.7	
					
L5				104.7 ± 3.1	97.1	279	259	17.7	
					
L6				97.7 ± 1.8	99.4	290	273	17.2	
					
L7				94.4 ± 0.8	97.1	266	243	17.7	
					
L8				117.8 ± 3.3	99.6	257	236	16.8	
					
L9				114.4 ± 2.5	99.4	279	259	16.3	
					
C1				119.4 ± 0.4	96.9	257	241	87	
					
C2	Carbonera (CPS)			122.8 ± 0.7	100	265	244	79	
					
C3				128.9 ± 5.9	100	252	229	85	
					
C4				111.1 ± 3.8	97.6	253	238	56	

MMC-PHA	OFMSW-WAS	Thermal drying	CHCl_3_ NaClO	75.2–100.9	75–100	269–303	252–278	7.0–20.2	Lorini et al. (2020)
	
MMC-PHA	OFMSW-WAS	Wet acidification	NaClO	95.8–97.5	99–100	289–293	268–270	13–13.6		

MMC-PHA	WAS	Freeze-drying	CHCl_3_	–	–	275	–	42	Frison et al. (2015)

MMC-PHA	WAS	Drying	CH_3_CH_2_CH(OH)CH_3_	–	–	291	–	26–34	Morgan-Sagastume et al. (2015)

MMC-PHA	Molasses	–	CHCl_3_	–	–	277–291	–	13–53	Bengtsson et al. (2010a)

MMC-PHA	Crude glycerol	Freeze-drying	Sonication + NH_3_	86	89	302–307	–	13	Burniol-Figols et al. (2020)

MMC-PHA	Dairy wastes	Freeze-drying	Non-ionic surfactants + DMC	57–92	78–90	242–255	–	9	Colombo et al. (2020)

*Thermal stabilized sample.

### Thermal Characterization

**FIGURE 1:**
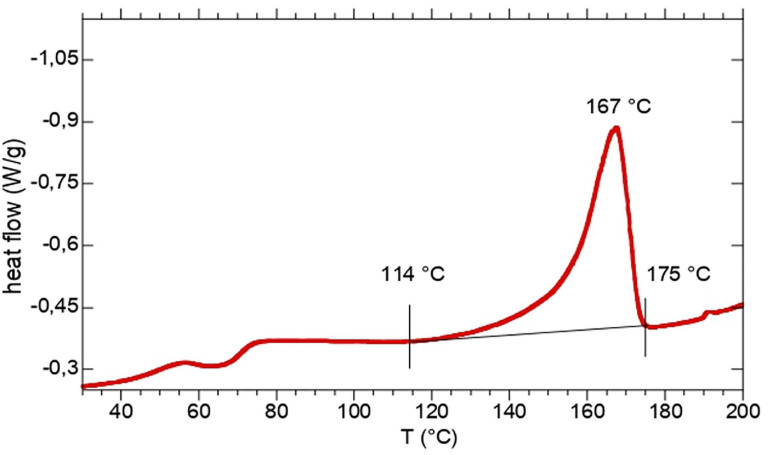
Single heating scan from DSC thermogram of sample TV-1.

Thermal characteristics of the extracted polymers, such as decomposition temperatures and related thermal stability, melting temperature and melting enthalpy have been determined through TGA and DSC. The results of the TGA experiments, including the PHA content (discussed in the previous section), the temperature at 10% decomposition (*T*_*d*_^10%^) and the temperature at maximum decomposition rate (*T*_*d*_^*max*^), are reported in Table 1, while melting temperature (*T*_*m*_) and melting enthalpy (Δ*H*_*m*_) from DSC are reported in Table 2. The comparison of the results showed that PHA thermal stability seems not to be affected by the feedstock used for PHA production. In fact, the average *T*_*d*_^10%^ and *T*_*d*_^*max*^ of extracted TV-samples were 265 ± 3°C and 288 ± 3°C while for L-samples the corresponding values were 257 ± 5°C (*T*_*d*_^10%^) and 276 ± 5°C (*T*_*d*_^*max*^) respectively. On the contrary, C-samples showed a lower thermal stability, with an average *T*_*d*_^10%^ and *T*_*d*_^*max*^ at 238 ± 4°C and 257 ± 3°C, respectively. The DSC profiles of sample TV1 is reported in Figure 1. The thermograms of the other TV samples did not remarkably change from that of TV1. In all the thermograms the main transition is the melting process occurring in the 160–175°C temperature range, but also a low temperature transition at about 60°C occurred.

**TABLE 2:** Thermal properties and crystallinity of PHA samples produced in Treviso and Carbonera pilot plants.

Sample	Feedstock	*T*_*m*_ (°C)	Δ*H*_*m*_ (J g^–1^)	χ_*c*_ (%)	*T*_*g*_ (°C)	References
TV1		167	41.8	28.6	–	
			
TV2		168	37.3	25.5	–	
			
TV3		171	47.7	32.7	–	
			
TV4		168	28.1	19.2	–	
			
TV5		168	38.2	26.1	–	
			
TV6	Treviso (OFMSW-WAS)	169	39.9	27.4	–	
			
TV7		170	38.8	26.6	–	
			
TV8		168	37.9	25.9	–	This study
			
TV9		170	48.5	33.2	–	
			
TV10		168	44.5	30.5	–	
			
TV11		167	36.2	24.8	–	
			
TV12		168	43.1	29.5	–	

C1		104.5	102	–	–19	
			
C2	Carbonera (CPS)	94	64	–	–14.5	
			
C3		104.7	96	–	–22	
			
C4		83	36.3	–	–12.5	

MMC-PHA	OFMSW-WAS	157–169	26–45	30–42	–	Lorini et al. (2020)

MMC-PHA	WAS	144	24	–	–0.5	Frison et al. (2015)

MMC-PHA	Cheese Whey	144–163	–	–	–3; 1	Hilliou et al. (2016)
	
MMC-PHA	Olive Mill Wastewater	150	–	–	0		

MMC-PHA	Molasses	144–165	5–17	–	–10.3; –3.3	Bengtsson et al. (2010a)
	
MMC-PHA	Synthetic VFA mix	150–172	20–82	–	–14; 4.8		

MMC-PHA	Synthetic VFA mix	153	35	–	–12.8	Laycock et al. (2014a)

MMC-PHA	Crude glycerol	154	–	–	–6.8	Burniol-Figols et al. (2020)

MMC-PHA	Dairy wastes	146–147	13–51	9–34	–7.3; –5	Colombo et al. (2020)

On the other hand, C-samples showed a different thermal behaviour, indeed the melting process occurred in a wider range shifted at lower temperature (80–105°C). Moreover, for C-samples, the glass transition occurred after the cooling step and it ranges between −22 and −12.5°C.

**FIGURE 2:**
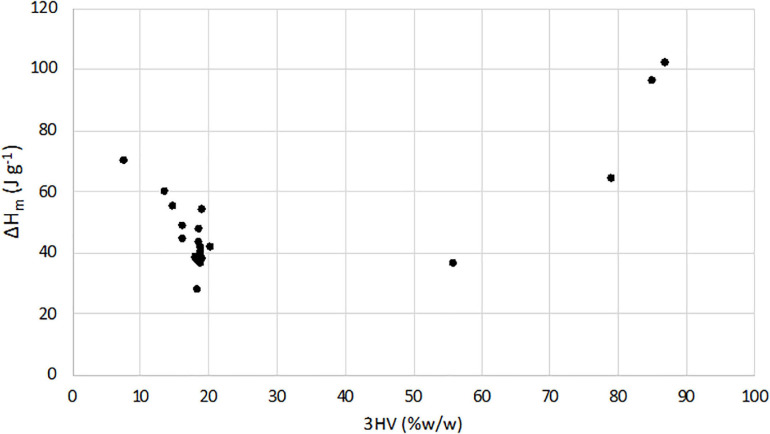
Relationship between Δ*H*_*m*_ (J g^− 1^) and monomeric composition (3HV content).

From the integrated melting enthalpy, the sample crystallinity was evaluated by Eq. 2 for TV-samples and then reported in Table 2. On the contrary, while there is adequate agreement between the results obtained from DSC for the P(3HB) homopolymer, in PHBV copolymers, it is quite difficult to measure a true crystallinity, particularly for copolymers with 3HV content higher than 20% w/w (Laycock et al., 2014b). Thus, in order to compare the melting behavior, the melting enthalpies (Δ*H*_*m*_) of TV and C-samples, determined by DSC, are reported as a function of 3HV content in Figure 2. Moreover, for a more complete evaluation of the 3HV content effects, results from a previous work (Lorini et al., 2020) are reported, too. In this way, it was possible to have a wider range of 3HV percentage, specifically between 7.7 and 87% w/w. The results clearly show that Δ*H*_*m*_ linearly increased with the 3HV content in the C-samples copolymer (3HV % w/w = 56-87), while it linearly decreased for the whole TV-samples (3HV % w/w = 7.7-20.2).

### Viscosity Average Molecular Weight

The determination of viscosity average molecular weight (*M*_*v*_) of each extracted sample gave different results for the three grades of collected material.

**FIGURE 3:**
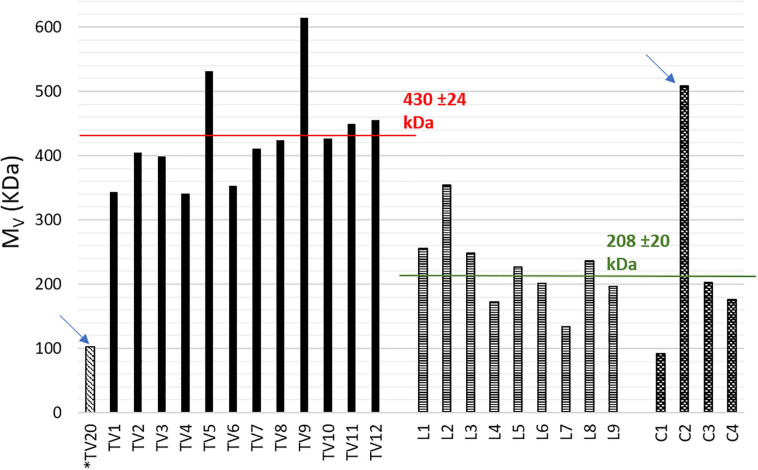
Comparison of viscosity average molecular weights (*M*_*v*_). Starred sample TV20 is thermal stabilized.

The results, reported in Figure 3, clearly appeared divided in two main groups: TV-samples and L and C-samples, with average values of 430 ± 24 and 208 ± 20 kDa, respectively, and one outlier for each group (102 and 508 kDa, respectively, for TV20 and C2).

### PCA Analysis

**FIGURE 4:**
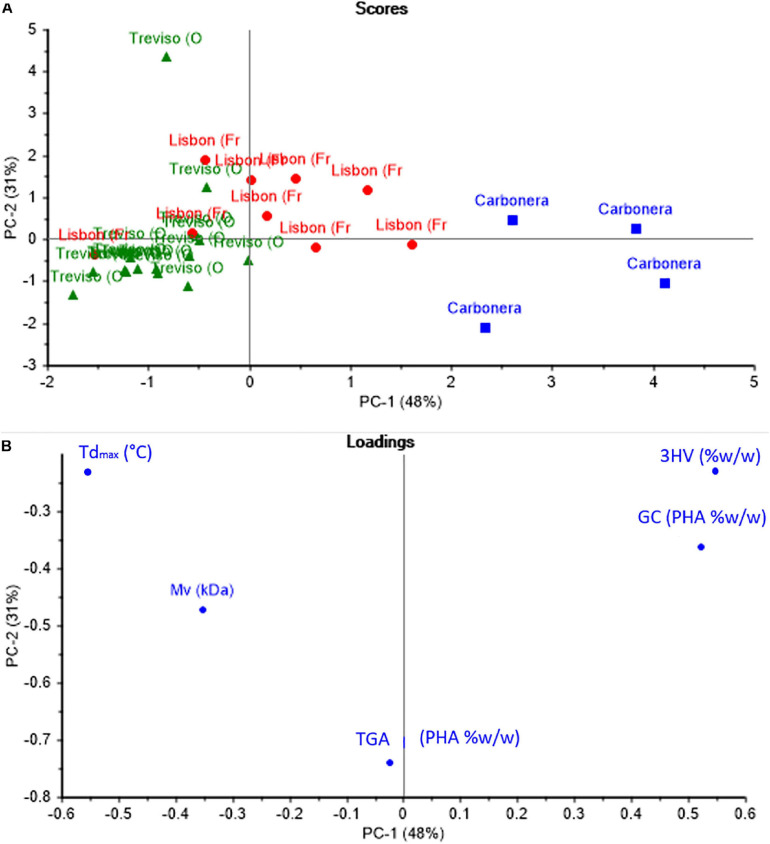
Complete PCA. **(A)** Scores plot. **(B)** Loadings plot.

Principal component analysis was defined as an effective statistical method used to establish relationships between different observations, such as similarities and inequalities. According to the PCA results, the first principal component (PC1) accounted for 48% of the total variance, 31% for the 2nd component (PC2). Figure 4A shows the score plot graphic and Figure 4B shows the loading plot graphic for PC1 and PC2 in PHA samples. When Figures 4A and B are evaluated together, analysis of the data obtained from the complete characterization of the PHA samples with the PCA allowed grouping the PHA samples into three groups to express and demonstrate their similarity and differences.

## Discussion

### Purity and Monomeric Composition

Recovery procedures for PHA from MMC, including non-chlorinated solvents extraction and disruption of NPCM through chemical or oxidizing agents, were widely investigated in the last decade as alternatives to traditional chloroform extraction (Werker et al., 2012; Villano et al., 2014; Samorì et al., 2015).

More recently, disruption of NPCM was obtained by using non-ionic surfactants as pretreatment before PHA extraction with DMC (Colombo et al., 2020) and by treating the biomass with ammonia solutions at high temperatures (Burniol-Figols et al., 2020). In a previous study (Lorini et al., 2020), NaClO digestion was investigated for PHA recovery from MMC produced at pilot scale. More in detail, PHA was produced in the pilot plant operating in Treviso, exploiting the same feedstock (OFMSW-WAS) and operative conditions and applying the same biomass stabilization methods described in this work. In this study, the effects of the extraction method optimized by Biotrend S. A. were evaluated on PHAs recovered from different biomasses. The extraction has been conducted on the wet acid stabilized centrifuged biomass for the whole set of PHA sample, except for sample TV20. Indeed, the latter was extracted from a thermal-stabilized dry biomass, produced during the first operative year of the pilot platform together with the samples analyzed elsewhere (Lorini et al., 2020). Results of the latter study are reported in Table 1 for a comparison. As explained in section “Purity and Monomeric Composition of Results,” sample TV20 had a lower purity, comparable with those thermal dried determined in the reference work by Lorini et al. (2020), than the rest of the whole set. Compared to other reference studies, in which NPCM disruption was conducted, the extraction method discussed in this work shows better performances in terms of polymer purity (higher than 92.0% w/w), in fact using NH_3_ (Burniol-Figols et al., 2020) or a combination of non-ionic surfactants and DMC (as PHA solvent) (Colombo et al., 2020) allowed to reach a PHA purity of 86 and 57 − 92% w/w, respectively. This evidence suggests that the optimized extraction method can be considered effective on all the types of biomass, in fact the same results were obtained independently from the PHA origin, that is kind of feedstock and operative conditions.

### Thermal Characterization

The purity obtained by GC-FID was confirmed through TGA analysis, in fact the polymer content in the samples was estimated from the main weight loss occurring between 250 and 320°C, as reported in literature (Hahn and Chang, 1995).

Moreover, in terms of thermal stability, the C-samples, with the highest 3HV content (56-87% w/w), showed the lowest degradation temperatures (on average *T*_*d*_^10%^ and *T*_*d*_^*max*^ at 238 ± 4°C and 257 ± 3°C, respectively). On the other hand, samples TV20 and L1, L2, L3, L4, with the lowest 3HV content (7.6% w/w) gave non-homogeneous results, indeed TV20 exhibited the highest *T*_*d*_^10%^ (289°C) and *T*_*d*_^*max*^ (307°C), while L-samples had lower values. Hence, the variability of the reported available data (Table 1) did not allow to enunciate a statement on the possible effect of monomeric composition on thermal stability.

Overall, such values indicated the high thermal stability of the polymer, comparable to those observed in other studies on MMC-PHA (Table 1) and on commercial P(3HB-co-3HV) (*T*_*d*_^*max*^ = 256–290°C; Morgan-Sagastume et al., 2015). DSC analysis allowed to evaluate the thermal behaviour of the three grades PHA. The melting temperature determined for TV-samples, ranging in 130–175°C, resulted comparable or higher than those reported in literature (Table 2). The low temperature exothermic transition at about 60°C could be considered as a cold crystallization (Figure 1), probably due to a partial crystallization started at low temperature during the extraction process (Wellen et al., 2015). Different thermal properties were found for PHA from Carbonera, in fact C-samples presented a lower *T*_*m*_ than TV and L-samples, and a *T*_*g*_ comparable or even lower than those reported in previous studies (Table 2). In fact, Bengtsson et al. (2010b) reported a *T*_*g*_ of −10.3°C and −14°C for PHA with 53 and 63% mol 3HV, produced from molasses and synthetic VFA solution, respectively. On the other hand, Serafim et al. (2008b) characterized a PHA, produced with a synthetic feedstock and having a 3HV content of 72% mol, which presented a *T*_*m*_ of 99.2°C and a *T*_*g*_ of −29.6°C, more similar to those of C-samples. For TV-samples, the determination of melting enthalpy allowed to calculate the crystallinity and to confirm the semicrystalline behavior of copolymer P(3HB-co-3HV) (Bengtsson et al., 2010a). Indeed, TV-samples are characterized by a χ_*c*_ (%) comparable to that reported in the previous work of Lorini et al. (2020) and in a study from Colombo et al. (2020) (Table 2). As reported in literature, it is quite difficult to determine the crystallinity of PHBV copolymers having 3HV content higher than 20%, since the melting process is affected by the presence of amorphous regions (Laycock et al., 2014b). Thus, Figure 2 shows a typical trend of Δ*H*_*m*_ as a function of 3HV content, indeed, as reported in Laycock et al. (2014b), a minimum in the melting enthalpy at 30–55% HV occurs.

Overall, these results highlight that the PHA samples, obtained with different process configuration and different feedstock, did not differ in thermal behaviour from other MMC-PHAs, independently from the kind of substrate exploited.

### Viscosity Average Molecular Weight

As mentioned in section “Viscosity Average Molecular Weight” of Results, Figure 3 shows the results of *M*_*v*_ determination. They are divided in two main groups having average values of 430 ± 24 and 208 ± 20 kDa.

To better understand the reason of this marked difference, it is useful to make a comparison between TV-samples of the present work and those of Lorini et al. (2020) (same pilot plant, feedstock, and stabilization methods).

As a main result of the previous work, the average *M*_*v*_ values of thermal dried samples were 127 ± 16 and 132 ± 10 kDa for NaClO- and CHCl_3_-extracted samples, respectively. Hence, no effects of the extraction step were found. Indeed, chloroform extraction was considered as a benchmark and it was concluded that NPCM disruption had not damaged the polymer. In this sense, we can consider NaClO extraction of the previous work as a benchmark, because it was applied on biomasses obtained in the same operative periods and with the same operative conditions. More in detail, sample TV-20 was thermally stabilized and obtained in parallel with thermal dried samples of the previous work, while samples TV-1-12 were acid stabilized and obtained in parallel with two acid stabilized samples of the previous work. These latter had a significantly higher *M*_*v*_ (370 and 424 kDa) than that obtained from thermal dried samples.

Hence, the *M*_*v*_ determination has allowed to evidence any possible effects of the biomass pre-treatment on the PHA and the effective capacity of acidification to stabilize PHA for downstream processing without affecting its molecular weight was confirmed (Werker et al., 2012). This evidence can be further confirmed by the results obtained in the present study. In fact, the same trend was found for sample TV-20 (*M*_*v*_ = 102 kDa) and TV-1-12 (341-615 kDa). Moreover, a study on PHB produced from crude glycerol and MMC reports significantly different *M*_*w*_ values for PHB after drying at 65°C and after lyophilization, respectively, 84-195 and 200-380 kDa. The authors justified the *M*_*w*_ decrease after drying with the occurrence of a partial hydrolysis due to heating (Hu et al., 2013). Besides, it can be concluded that the extraction method applied by Biotrend S. A. had no negative effects on PHAs chains, since the results of TV-samples are similar those obtained from the benchmark NaClO extraction (Lorini et al., 2020). On the other hand, L and C-samples showed a significantly lower *M*_*v*_, ranging between 134-354 and 92-203 kDa with C2-sample as an outlier (508 kDa). Taking into account that the stabilization method applied on PHA-rich biomass at the end of the accumulation and the extraction method are the same for all the three grades PHA (TV, L, and C-samples), it is possible to infer that the marked difference in *M*_*v*_ is probably due to the different feedstocks and operative conditions applied in the pilot plants. According to what has been reported in recent studies of MMC-PHA produced from wastes and/or synthetic VFA mixture there is no correlation between the *M*_*w*_ and the 3HV content in the copolymer (Frison et al., 2015; Hilliou et al., 2016). Literature data on commercial polymers from pure culture report that molecular weight (*M*_*w*_) ranges in 200–660 kDa (Morgan-Sagastume et al., 2015; Colombo et al., 2017). On the contrary, it is often reported that MMC are able to produce PHA with even higher *M*_*w*_, comparable to the highest *M*_*v*_ determined for TV-samples. Previous studies reported *M*_*w*_ of 340–540 kDa (Villano et al., 2014), 440–630 kDa (Laycock et al., 2014a) for PHA produced from synthetic VFA mixture; 400–600 kDa (Burniol-Figols et al., 2020) from crude glycerol fermentation liquid; 650 kDa (Frison et al., 2015) from WAS; 800 kDa (Colombo et al., 2017) from percolates of the OFMSW; 800 kDa (Colombo et al., 2020) from dairy wastes. The cited works have been conducted at laboratory scale, while a study from full-scale is noteworthy and it reported a PHA from activated sludge with *M*_*w*_ of 400–500 kDa (Arcos-Hernández et al., 2015; Werker et al., 2018).

### PCA Analysis

Considering Figures 4A and B together, analysis of the data obtained from the complete characterization of the PHA samples with the PCA allowed grouping the PHA samples into three groups to express and demonstrate their similarity and differences. According to that discussed in the previous sections, it is possible to conclude that the main differences among the three grades PHA are due to the different feedstocks and pilot plants, with more remarkable evidence in C-samples. Indeed, PC1 presents a direct correlation with 3HV content that is determining for separate C-samples as a single group, since 3HV is even higher than 80% w/w, namely the opposite composition of TV and L-copolymer. Loadings plot (Figure 4B) shows that *M*_*v*_ is also a relevant parameter, in fact TV and L-samples are separated mainly because of the marked difference in *M*_*v*_ values and the two groups are shown in scores plot (Figure 4A).

### Potential Applications and Further Scenario

Polyhydroxyalkanoates produced in Treviso showed *M*_*v*_ in line with values for good mechanical properties, required for thermoplastic applications (Laycock et al., 2014b). Hence the PHA produced in Treviso pilot plant deserves further investigation. Up to now, tests for future applications evaluated the possibility of compounding PHA from OFMSW-WAS and other biodegradable polymers, as PBS (polybutylene succinate) or PBAT (polybutylene adipate terephthalate). Preliminary results showed that it is possible to obtain biodegradable films (for agricultural purposes) with acceptable mechanical and permeability properties through melt compounding and blown extrusion, starting from mixtures at different PHA content. Also packaging applications have been explored by Melendez-Rodriguez et al. (2020) on PHA from Treviso, carrying out a wide characterization on fiber-based continuous films obtained starting from electrospun PHA. The combination of electrospinning and a mild annealing postprocessing below the biopolymer’s *T*_*m*_ was able to yield unique biomaterials with balanced mechanical, thermal and barrier properties (Melendez-Rodriguez et al., 2020). Furthermore, one of the main concerns relating to the use of waste as feedstock is the content of pollutants that can migrate from the waste, through the technology chain, and into the end-products. PHAs from Treviso and Lisbon were analyzed in order to determine the presence of relevant contaminants, such as heavy metals (Astolfi et al., 2020) and polychlorinated biphenyls (PCB) (Riccardi et al., 2020). The total content of the analyzed metals and PCBs for all tested PHA types complied with the current regulations and guidelines. Therefore, the use of such waste as raw material for PHA production process appears to be safe for the environment and human health. As an added value, PHA characterized by low *M*_*v*_ or purity may be exploited as electron donor for biological reductive dechlorination, applied into permeable reactive barriers (PRBs) for groundwater remediation from chlorinated hydrocarbons (Baric et al., 2014).

## Conclusion

This study showed the possibility to exploit different organic biowaste as raw material for biodegradable polymers, characterized by thermal and chemical properties comparable to commercial plastics. Indeed, the application of a stabilization method at the end of the accumulation step followed by an efficient extraction allowed to reach high purity of the end-product and to preserve the polymer properties, mostly the molecular weight. This latter was influenced by the type of feedstock, indeed on equal terms of monomeric composition with PHA from FW, a PHA with significantly higher *M*_*v*_ was produced from OFMSW-WAS. Monomeric composition was strongly influenced by the VFAs composition and the operative conditions, since C-samples have an opposite 3HV content (56–87% w/w) than TV and L-samples. Thermal properties are all in the same range for the whole samples set and comparable to those reported in literature. Moreover, melting enthalpy is dependent from 3HV content and the showed trend complies with that reported in literature. As a conclusion, the present study underlines that, from three different processes at pilot scale, different PHAs having adjustable properties can be produced with a long time stability. The real novelty of the work is to show how waste-derived biopolymers can achieve good characteristics and workability, and this information has a pivotal role in future development and optimization of these processes, including the kind of downstream and applications.

## Data Availability Statement

The raw data supporting the conclusions of this article will be made available by the authors, without undue reservation.

## Author Contributions

LL: investigation, data curation, and writing – original draft. AM: supervision and visualization. GC: formal analysis. NF and BSF: resources. MR: resources and funding acquisition. MV: visualization. MM: funding acquisition and project administration. FV: conceptualization, data curation, writing – original draft, and supervision. All authors contributed to the article and approved the submitted version.

## Conflict of Interest

BSF was employed by company Biotrend SA. The remaining authors declare that the research was conducted in the absence of any commercial or financial relationships that could be construed as a potential conflict of interest.
